# Distribution and Antimicrobial Susceptibility Pattern of Bacterial Pathogens Causing Urinary Tract Infection in Urban Community of Meerut City, India

**DOI:** 10.1155/2013/749629

**Published:** 2013-10-29

**Authors:** Devanand Prakash, Ramchandra Sahai Saxena

**Affiliations:** Department of Botany, Meerut College, Meerut, Uttar Pradesh 250 001, India

## Abstract

Urinary tract infection is one of the common infections in the Indian community. Distribution and susceptibility of UTI-causing pathogens change according to time and place. This study was conducted to determine the distribution and antimicrobial susceptibility of uropathogens in the Indian community as well as to determine the effect of gender and age on the etiology of bacterial uropathogens. Clean catch midstream urine samples were collected from 288 patients of the age ranging from 15 to ≥48 years. Antimicrobial susceptibility was performed on all isolated bacteria by Kirby Bauer's disc diffusion method. The multiple antibiotic resistance (MAR) index of each antibiotic was calculated. The UTI prevalence was 53.82% in patients; however, the prevalence was significantly higher in females than in males (females: 73.57%; males: 35.14%; *P* = 0.000). Females within the age group of 26–36 years and elderly males of ≥48 years showed higher prevalence of UTI. Gram negative bacteria (90.32%) were found in high prevalence than Gram positive (9.68%). *Escherichia coli* (42.58%) was the most prevalent gram negative isolate. Nitrofurantoin (78.71%) was found the most resistant drug among all uropathogens. Tested carbapenems were found the most susceptible drug against isolated uropathogens which showed 92.26% and 84.52% susceptibility, respectively.

## 1. Introduction

Urinary tract infection (UTI) is the commonest bacterial infectious disease in community practice with a high rate of morbidity and financial cost. It has been estimated that 150 million people were infected with UTI per annum worldwide which costing global economy more than 6 billion US dollars [1]. UTIs is described as a bacteriuria with urinary symptoms [2]. UTI can affect lower and sometimes both lower and upper urinary tracts. The term cystitis has been used to define the lower UTI infection and is characterized by symptoms such as dysuria, frequency, urgency, and suprapubic tenderness. The presence of the lower UTI symptoms does not exclude the upper UTI which is often present in most UTI cases [3]. The treatment of UTI can be classified into uncomplicated and complicated on the basis of their choice of treatment [4]. UTI is more common in females than in males as female urethra structurally found less effective for preventing the bacterial entry [5]. It may be due to the proximity of the genital tract and urethra [6] and adherence of urothelial mucosa to the mucopolysaccharide lining [7]. The other main factors which make females more prone to UTI are pregnancy and sexual activity [8]. In pregnancy, the physiological increase in plasma volume and decrease in urine concentration develop glycosuria in up to 70% women which ultimately leads to bacterial growth in urine [9]. Also in the nonpregnant state the uterus is situated over the bladder whereas in the pregnant state the enlarged uterus affects the urinary tract [10]. Sexual activity in females also increases the risk of urethra contamination as the bacteria could be pushed into the urethra during sexual intercourse as well as bacteria being massaged up the urethra into the bladder during child birth [11, 12]. Using a diaphragm also causes UTI as it pushes against the urethra and makes the urethra unable to empty the bladder completely and the small concentration of urine left in the bladder leads to the growth of bacteria which ultimately causes UTI [13]. 

The spectrum of bacteria causing complicated UTI is much broader than of those causing uncomplicated UTI. However, the most commonly encountered microorganisms are Gram negative bacteria including *Escherichia coli, Citrobacter *spp., *Enterobacter aerogenes, Pseudomonas aeruginosa, *and *Proteus vulgaris *whereas *Klebsiella *spp., *Staphylococcus aureus, *and *Salmonella *spp. are found rarely [14].

Increasing multidrug resistance in bacterial uropathogens is an important and emerging public health problem. The Infectious Disease Society of America (IDSA) identified some microorganisms for new effective therapies. Those microorganisms were called “ESKAPE pathogens” which include *Enterococcus faecium, S. aureus, Klebsiella *spp., *Acinetobacter *spp., *Pseudomonas *spp., and *Enterobacter *spp. Increasing drug resistance in UTI needs regular monitoring of the antibiotic susceptibility of uropathogens in a particular area. Various factors such as the type of UTI (complicated or uncomplicated), gender, age, and previous history of antibiotic therapy of each UTI patient should also be considered to find out the correct global data on susceptibility [15]. The distribution of antimicrobial susceptibility data of UTI-causing microorganisms changes from time to time and from place to place [13]. The susceptibility data provided by regional microbiology laboratories helps to choose the empirical choice of antimicrobials to treat UTI; however, these conditions are limited to complicated UTI as the samples of uncomplicated UTI are rarely sent to laboratories [16, 17]. Generally, the antimicrobial treatment is initiated before the laboratories results which may lead to the frequent misuse of antibiotics [18]. The resistance pattern of community acquired uropathogens has not been extensively studied in India [19–21]. To the best of our knowledge, no data regarding the bacterial resistance in UTIs from Meerut District (Uttar Pradesh), India, has been documented. Since most UTIs are treated empirically, the criteria for the selection of antimicrobial agents should be determined on the basis of the most likely pathogen and its expected resistance pattern in a geographic area. Therefore there is a need for periodic monitoring of etiologic agents of UTI and their resistance pattern in the community. 

This study was undertaken in view of paucity of reports of UTIs in patients of Meerut District (Uttar Pradesh), India. The aim of the study is to determine the prevalence of UTI in male and female patients as well as the effect of gender and age on its prevalence. The UTI-causing microorganisms, their distribution among different ages and genders, and their antimicrobial susceptibility will also be determined.

## 2. Material and Methods

### 2.1. Study Area

The study was carried out in the microbiology laboratory of the Department of Botany, Meerut College, Meerut (Uttar Pradesh), India. The urine samples were collected from the OPDs (outpatients departments) section of three major hospitals (Meerut Kidney Hospital, Pyarelal Hospital, and Jaswant Rai Hospital) of Meerut city. These sample collection sites were chosen as they mostly covered the urban area of the city. The duration of the study was one and a half year from July 2011 to January 2013.

### 2.2. Study Population

The urine samples of 288 patients, comprised of 148 males and 140 females, who attended the outpatient departments (OPDs) of three hospitals and had clinical evidence of urinary tract infection, determined by treating physicians, were included in this study. The age of patients included in the study ranged from 15 to ≥48 years. Patients with history of hospital admission a week before their presentation in OPDs were excluded from the study to rule out hospital-acquired infections. The patients on antibiotic therapy were also excluded from the study. 

### 2.3. Sample Collection

Clean catch midstream urine was collected from each patient into a 20 mL calibrated sterile screw-capped universal container which was distributed to the patients. The specimens were labeled, transported to the laboratory, and analyzed within 6 hours. In each container boric acid (0.2 mg) was added to prevent the growth of bacteria in urine samples. All patients were well instructed on how to collect sample aseptically prior to sample collection to avoid contaminations from urethra. Verbal informed consent was obtained from all patients prior to specimen collection. The study was conducted after due ethical approval which was subjected to the hospital administrations.

### 2.4. Sample Processing

A calibrated loop method was used for the isolation of bacterial pathogens from urinary samples. A sterile 4.0 mm platinum wired calibrated loop was used which delivered 0.001 mL of urine. A loopful urine sample was plated on Cystine-Lactose-Electrolyte Deficient (CLED) agar, MacConkey agar, and blood agar medium (Hi Media Laboratories, Mumbai, India). The inoculated plates were incubated at 37°C for 24 h and for 48 h in negative cases. The number of isolated bacterial colonies was multiplied by 1000 for the estimation of bacterial load/mL of the urine sample. A specimen was considered positive for UTI if an organism was cultured at a concentration of ≥10^5^ cfu/mL or when an organism was cultured at a concentration of 10^4^ cfu/mL and >5 pus cells per high-power field were observed on microscopic examination of the urine [22]. 

### 2.5. Identification and Maintenance of Pure Bacterial Isolates

Identification of bacterial isolates was done on the basis of their cultural and biochemical characteristics. Gram negative bacteria were identified by the standard biochemical tests [14, 23] and Gram positive microorganisms were identified with the corresponding laboratory tests: catalase, coagulase, and mannitol test for *Staphylococcus aureus *[24]. Identified and pure isolates were maintained in nutrient agar slants and incubated at 37°C for 24 hrs. The isolates were subcultured periodically.

### 2.6. Antibiotic Susceptibility Testing

Isolates were tested for antimicrobial susceptibility testing by the standard Kirby Bauer's disc diffusion method [25]. Standard inoculums adjusted to 0.5 McFarland was swabbed on Mueller Hinton agar and was allowed to soak for 2 to 5 minutes. After that antibiotic disks were placed on the surface of media and pressed gently. Mueller Hinton agar plates were then incubated at 37°C for 24 h. After 24 h the inhibition zones were measured and interpreted by the recommendations of clinical and laboratory standards [26]. The following standard antibiotic discs were used for the isolates, ciprofloxacin (CIP), moxifloxacin (MOX), ofloxacin (OFL), sparfloxacin (SPR), levofloxacin (LEV), nalidixic acid (NAL), gatifloxacin (GTX), tobramycin (TOB), amikacin (AMK), gentamycin (GET), ceftazidime (CTZ), cefotaxime (CTX), ceftriaxone (CFX), imipenem (IMP), meropenem (MRP), nitrofurantoin (NTF), netillin (NTL) and co-trimoxazole (COT). Standard strains of *E. coli* (ATCC 25922), *S. aureus* (ATCC 25923), and *P. aeruginosa* (ATCC 27853) were used routinely in this study as control. The mean of triplicates was considered and standard error of mean was calculated by Microsoft Excel ver. 2007. 

### 2.7. Multiple Antibiotic Resistance (MAR) Indexing

The multiple antibiotic resistance indices (MARI) were calculated by the method described by Tambekar et al. [18]. The following formula was used for the calculation of MAR index of antibiotics: 

MAR index for an antibiotic = [number of antibiotics resistant to the isolates/(number of antibiotics × Number of isolates)]. The number of MAR index for an antibiotic indicates its sensitivity and resistance. Antibiotic resistance increases with the increasing MAR values.

### 2.8. Statistical Analysis

The data were analyzed using Chi-square (*χ* ^2^) test, confidence interval (CI), odds ratio (OR) analysis, and student's *t*-test for paired samples. Relative risk and odds ratio were performed to compare the risk factors in the different groups of interest (male and female patients), and the Chi square test was conducted to find out the significant difference between the isolated uropathogens, infected male and female patients related to different age groups, and statistical comparisons for the MAR indices group; however, *χ* ^2^ test for trend was conducted for antimicrobial resistance and sensitivity variables among all isolated uropathogens. The paired *t*-test was used to compare resistance versus sensitivity against isolates. A *P* value of <0.05 was considered as statistically significant for all tests and at 95% level of confidence interval. All statistical tests were performed by Statistical Package for Social Sciences (SPSS) software, Inc. 233 South Wacker Drive, 11th Floor Chicago, IL 60606-6412, USA, for Windows, version 20. The *χ* ^2^ test for trend and graphs were prepared by GraphPad PRISM software (version 5.03), Inc. 2236 Avenida de la Playa La Jolla, CA 92037, USA.

## 3. Results

The overall prevalence of UTI in both male and female patients was found to be 53.82%. Total 155 urine samples showed the significant bacterial growth which were comprised of 52 (35.14%) samples from males and 103 (73.57%) from females. These results indicated that the prevalence of UTI was higher in female patients than in males. The *P* value and the odds ratio showed a the significant variation between male and female patients (Table 1). 

**Figure 1:**
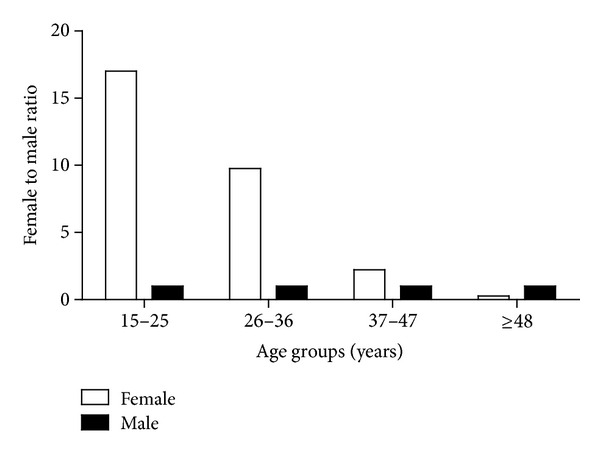
Female to male ratio for the occurrence of UTI.

The highest susceptible age group of patients to UTI was ≥48 years (63.51%) followed by 26–36 years (58.11%), 15–25 years (54.55%), and 37–47 years (39.19%). Comparatively, however, more cases of UTI were observed in females than in males in all age groups. The highest prevalence of UTI in females was found in the age group of 26–36 years (90.69%); however in males the highest susceptible age group to UTI was ≥48 years (71.15%). The Chi square test showed statistically significant variations (*P* < 0.05) at 95% level of confidence interval for the infected and not infected male and female patients variables among all age groups. For the infected and not infected male patients variable the Chi-square test values were *χ* ^2^ = 13.081; degree of freedom = 1; *P* = 0.000 and the values for infected and not infected female patients were *χ* ^2^ = 31.114; degree of freedom = 1; *P* = 0.000 (Table 2). The highest female to male ratio for the occurrence of UTI was found in the age group of 15–25 years (17 : 1) followed by 26–36 years (9.75 : 1), 37–47 years (2.22 : 1), and ≥48 years (0.27 : 1). The *χ* ^2^ test for trend results showed significant variations (*P* < 0.05) between the female to male ratio variables in all age groups at 95% confidence interval level (*χ* ^2^ = 5.228; degree of freedom = 1; *P* = 0.0222) (Figure 1).

A total of 155 bacterial uropathogens comprised of 140 (90.32%) Gram negative and 15 (9.68%) Gram positive were isolated from positive urine samples. *Escherichia coli *was found the dominant bacteria among all isolated uropathogens with the prevalence rate of 42.58%. The second most prevalent isolate was *Klebsiella pneumoniae *(18.71%) followed by *Pseudomonas aeruginosa *(12.90%), *Staphylococcus aureus *(9.68%), *Proteus *spp. (9.03%), and *Enterobacter* spp. (7.10%). There was no statistically significant variation (*P* > 0.05) was found among the isolates (Table 3). Out of 140 Gram negative bacteria 50 (35.71%) were isolated from males and 90 (64.29%) were from female patients. Only 2 (13.33%) gram positive bacteria were isolated from male and 13 (86.67%) were isolated from female patients. The highest number of gram positive and negative uropathogens (39) was found in the female patients of the age group 26–36 years followed by 37 uropathogens which were isolated from the male patients with the age group of ≥48 years (Table 4). 

**Figure 2:**
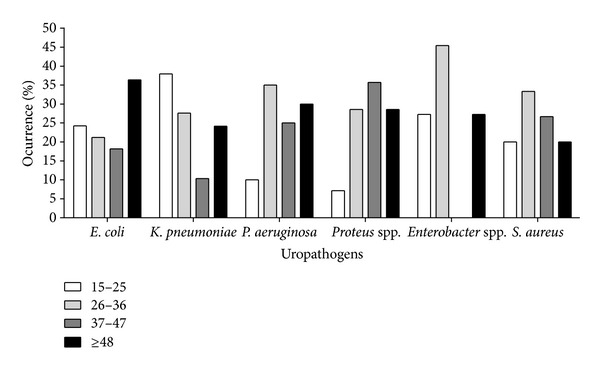
Frequency distribution of uropathogens between different age groups.

The highest to lowest prevalence rate for the occurrence of different isolated uropathogens within the age groups were as follows: *E. coli*—≥48 years (36.36%); 15–25 years (24.24%); 26–36 years (21.21%); 37–47 years (18.18%): *K. pneumoniae*—15–25 years (37.93%); 26–36 years (27.59%); ≥48 years (24.14%); 37–47 years (10.34%): *P. aeruginosa*—26–36 years (35.00%); ≥48 years (30.00%); 37–47 years (25.00%); 15–25 years (10.00%): *Proteus *spp.—37–47 years (35.71%); ≥48 years and 26–36 years (28.57%); 15–25 years (7.14%): *Enterobacter *spp.—26–36 years (45.45%); ≥48 years and 26–36 years (27.27%); 37–47 years (0.00%): *S. aureus*—26–36 years (33.33%); 37–47 years (26.67%); ≥48 years and 15–25 years (20.00%) (Figure 2). 

**Figure 3:**
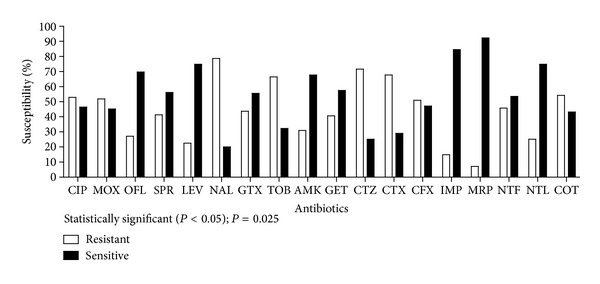
Overall resistance and sensitivity of all isolated uropathogens against tested antibiotics.

Antibiotic susceptibility results showed the resistant and susceptible antibiotics for the tested uropathogens. Overall NAL was found the most resistant drug as 122 (78.71%) uropathogens were found resistant against NAL. The second most resistant drug was CTZ (71.61%) followed by CTX (67.74%); however, the most sensitive drug against all uropathogens was MRP (92.26%) followed by IMP (84.52%), LEV, and NTL each showing 74.84% sensitivity (Figure 3). The *χ* ^2^ test for trend results showed a statistically significant variation (*P* < 0.05) between the resistant and sensitive variables (*χ* ^2^ = 9.152; degree of freedom = 1; *P* = 0.0025). 

TOB was found the highest resistant drug against 96.97% *E. coli *followed by NAL (90.91%) and CTX (87.88%); however, both carbapenems IMP and MRP showed the highest sensitivity against 98.45% and 95.45% *E. coli*. 79.31% of *K. pneumoniae *were resistant against CTZ and LEV was found the most susceptible drug with the rate of 89.66%. In case of *P. aeruginosa *the highest resistant and susceptible antibiotics were SPR (100%), and MRP (100%) respectively. 92.86% of tested *Proteus *spp. were resistant against CFX and 100% sensitive against both carbapenems (IMP and MRP). *Enterobacter* spp. showed 81.82% resistance against NTF; however, all (100%) were sensitive to OFL, SPR, LEV, IMP, and MRP. All *S. aureus* (100%) showed resistance against NAL and CTX; however, IMP was found 100% sensitive followed by SPR, CFX, and NTL (each showed 93.33% sensitivity against *S. aureus* isolates) (Table 5). The results of the paired *t*-test showed that there was no statistical significance between *E. coli* resistant versus sensitive variables (*P* = 0.876), *K. pneumoniae *resistant versus sensitive variables (*P* = 0.232), *P. aeruginosa *resistant versus sensitive variables (*P* = 0.950), *Proteus* spp. resistant versus sensitive variables (*P* = 0.162) and *S. aureus* resistant versus sensitive variables (*P* = 0.072), however, *Enterobacter* spp. showed the significant variations between resistant versus sensitive variables (*P* = 0.000). 

The highest MAR index was found for NAL (0.044) followed by CTZ (0.039) and CTX (0.038) indicating that these antibiotics were highly resistant among all tested uropathogens; however, the lowest MAR index was found for both carbapenems MRP and IMP which were 0.004 and 0.008, respectively, indicating the highest sensitivity against uropathogens. The *χ* ^2^ test results showed no statistically significant variation among the MAR indices of all tested antibiotics (*χ* ^2^ = 1.556; degree of freedom = 15; *P* = 1.000). 

## 4. Discussion

This study provides valuable data to compare and monitor the status of antimicrobial resistance among uropathogens to improve efficient empirical treatment. Increasing antimicrobial resistance has been documented globally [27–33]. The prevalence of UTI was found to be 53.82% in this study and this rate of prevalence is higher than in the other studies which accounts for 25.6% [34], 22% [35], 38.6% [36], 35.5% [11], 4.2% [37], 17.19% [20], 10.86% [21], 34.5% [38], and 36.68% [39] in India; however, the prevalence rate of UTI in our study correlates with other studies done in South Trinidad [40], and in the Mexican population [41] which showed such more highly significant uropathogens 49% and 97.3%, respectively.

Our study showed a high prevalence of UTI in females (73.57%) than in males (35.14%) which correlates with other findings which revealed that the frequency of UTI is greater in females as compared to males [6, 30, 40–44]. The reason behind this high prevalence of UTI in females is due to close proximity of the urethral meatus to the anus, shorter urethra, sexual intercourse, incontinence, and bad toilet [45–47]. 

The occurrence of UTI recorded among the elderly (≥48 years, 63.51%) compared to young age patients (26–37 years, 58.11%; 15–25 years, 54.55%) and middle-age patients (37–47 years, 39.19%) in this study differs from the other studies done in Kuwait [48] and Nigeria [49] in which the highest incidence of UTI was recorded among the age group 20 to 50 years (63.4 and 74.7%, resp.) and lowest among the age group >50 years (13.3 and 10.3%, resp.). However, our results agree with the study done in Japan with a 20-year period in which a trend of increasing complicated UTI was reported in elderly patients [50]. In our study it was found that the elderly males (≥48 years) had a higher incidence of UTI (71.15%) when compared with the elderly females (45.45%). This finding is similar to a study conducted at a tertiary care hospital in Jaipur, Rajasthan, India [44]. The main cause behind this increasing incidence of UTI with advancing age in males is due to prostate enlargement and neurogenic bladder [51]. This factor is also reported by other authors whose studies showed that the prostate disease in males is responsible for the increase in incidence of UTI and decrease in female : male ratio in patients above 50 years [52]. 

Females of the age group 26–36 years were found more susceptible (90.69%) to UTI followed by 15–25 years (82.93%), 37–47 years (58.82%), and ≥48 years (45.45%). These findings correlate with other reports which showed that females are more prone to UTIs than males during adolescence and adulthood [12, 18, 20, 44, 53–58]. The factors of this increasing incidence of UTI in young age females are associated with high sexual activity, recent use of a diaphragm with spermicide, and a history of recurrent UTIs [59]. 

The highest incidence of UTIs among female to male ratio was found in the age group of 15–25 years (17 : 1) followed by 26–36 years (9.75 : 1), 37–47 years (2.22 : 1), and ≥48 years (0.27 : 1). These findings differ from other reports [57, 60] which stated a lower female to male ratio in neonates and young children. The prevalence rate of UTI in boys depends on many factors including congenital malformations and uncircumcised genitalia which are often contaminated [57]. 

In this study, the Gram negative bacilli constituted 90.32% of the total bacterial isolates while Gram positive cocci constituted 9.68%. *Escherichia coli *(42.58%) was found the most prevalent gram negative bacteria in the positive urine samples of UTI. This result is consistent with reports from other studies [38, 48, 49, 53, 61–63] but differs from the reports in which *P. aeruginosa *[64] and *Klebsiella *spp. [65] were recorded as the predominant bacteria in UTI. Other isolated bacteria from UTI cases in this study were *K. pneumoniae *(18.71%), *P. aeruginosa *(12.90%), *S. aureus *(9.68%), *Proteus *spp. (9.03%), and *Enterobacter *spp. (7.10%). These findings were not correlate with other reports in which *P. aeruginosa* was reported as the second most common bacterial isolate in UTI studies in India [18] and Lafia, Nigeria [12]; however, these results correlates with others in which *Klebsiella *spp. was reported as the second most frequently isolated organism in UTI [32, 54, 63, 66, 67]. 

The studies on UTI in other places of the world also showed that *E. coli *and *Klebsiella* spp. are the commonest uropathogens in UTI [20, 21, 68–70]. Higher incidence of gram negative bacteria, related to Enterobacteriaceae, in causing UTI has many factors which are responsible for their attachment to the uroepithelium. In addition, they are able to colonize in the urogenital mucosa with adhesins, pili, fimbriae, and P-1 blood group phenotype receptor [51].

In females of all age categories, *E. coli *is the most frequently isolated uropathogen which correlates with other studies [71–73] but not with others which found that *E. coli *causes most male UTIs, followed by other Enterobacteriaceae and Enterococci [74, 75] whereas *Proteus mirabilis *was more frequently isolated in the younger female patients of UTI and *K. pneumoniae *in the elderly patients [72].

Both carbepenems (MRP and IMP) used in this study were found to be the most sensitive drugs against all isolated uropathogens. The sensitivity rate of carbepenems among uropathogens was as follows: *E. coli* (MRP; 95.45% and IMP; 98.89%), *P. aeruginosa *(MRP; 100% and IMP; 95.00%), *Proteus *spp. (MRP; 100% and IMP; 100%), *Enterobacter *spp. (MRP; 100% and IMP; 100%), and *S. aureus *(MRP; 80% and IMP; 100%), followed by LEV and NTL each of which showed 74.84% sensitivity, however, *K. pneumonia* did not show a high susceptibility to IMP (24.14%) but it was susceptible to MRP (86.21%). These antibiotic susceptibility results correlate with other studies [76, 77]. Another study conducted in India showed that meropenem was highly sensitive against Gram negative bacilli whereas cephalosporin showed highest resistance against gram negative rods [78]. In other study, meropenem and imipenem were found to be 98% and 100% sensitive, respectively, against highly resistant gram negative bacilli [79]. A study done in King Fahd Hospital, Saudi Arabia showed that meropenem was 95.8% sensitive followed by amikacin (93.7%) and imipenem (91.71%) against extended spectrum *β* lactamase producing *E. coli* [80]. 

Tested fluoroquinolones in this study showed the highest resistance among uropathogens as in *E. coli*; NAL (90.91%): *K. pneumoniae*; CIP (79.31%), *P. aeruginosa*; SPR (100%), and *S. aureus*; NAL (100%); however, III generation cephalosporin showed the highest resistance in *K. pneumoniae*; CTZ (79.31%) the *Proteus *spp.; CFX (92.86%), and *S. arueus*; CTX (100%). This high rate of resistance against fluoroquninolones was also suggested by other studies done in Spain, Europe, and Iran [33, 81] and also by other studies done in India [21, 44, 82]. Another study done in Spain also showed the reduced susceptibility of *E. coli *isolates from patients with UTI to Fluoroquinolones (16%) [81]. This reduced susceptibility might be due to using antibiotics without restriction. In several studies it has been shown that the highly prescribing habits of the physicians are the driving factor for the antibiotic resistance for this group of antibiotic [83–85]. McEwen et al. [86] found that 37% of physicians actually prescribe trimethoprim-sulphamethoxazole closely followed by fluoroquinolones (32%) and the average duration of antibiotic therapy is 8.6 days in the United States which is the best example of this problem; empiric use of fluoroquinolones should be restricted and founding the strategies against increasing resistance of pathogens to these antibiotics should be done. 

Our finding about the Fluoroquinolones did not correlate with others which showed that they were highly effective (sensitive) [11, 55, 64, 87, 88]. For these organisms, drugs with inhibitors like Augmentin may be tried [89] but such drugs should be reserved for the last line of treatment. The alarming finding in this study is the resistance to third-generation cephalosporin; the highest resistance was seen against CTZ (71.61%) followed by CTX (67.74%) among all uropathogens. This is an indication that many of the organisms are ESBL producers [90]. The other possible explanation behind this situation is that the III generation cephalosporin has been in use for a long period and must have been abused and over time organisms have developed resistant mechanisms due to changing their mode of action. The inappropriate usage of wide spectrum antibiotics, insufficient hygiene, immunosuppression, and a prolonged stay in the hospital are some other major etiological factors that elevate the chances of MDR infections [89]. 

## 5. Conclusion

Against the background of paucity of reports of UTI in Meerut city (Uttar Pradesh), India, this is the first study conducted to determine the prevalence of UTI, the effect of gender and age on its prevalence, and their susceptibility profile in the community of Meerut city. This study provides valuable laboratory data to monitor the status of antimicrobial resistance among uropathogens and to improve treatment recommendations in a specific geographical region. The study also allows comparison of the situation in Meerut with other regions within and outside the state as well as in the country. 

## Figures and Tables

**Table 1:** Distribution of significant and nonsignificant growth pattern of screened urinary samples.

Gender	Tested	Urine samples				Odds ratio			Relative risk			Pearson Chi-square value	*P* value
		No significant growth (<10^5^ cfu/mL)		Significant growth (>10^5^ cfu/mL)		Value	95% CI		Value	95% CI			
		*N*	%	*N*	%		Lower	Upper		Lower	Upper		
Male	148	96	64.86	52	35.14	0.195	0.000	0.322	0.478	0.376	0.607	42.764; df = 1	0.000^†^
Female	140	37	26.43	103	73.57								
Total	288	133	46.18	155	53.82								

*N*: number; %: percentage; CI: confidence interval; df: degree of freedom; ^†^significant.

**Table 2:** Prevalence of UTI in different age groups and genders.

Age group (in years)	Males			Females			Total number of males and females	Number of not infected males and females (%)	Number of infected males and females	UTI percentage
	Total	Infected (%)	Not infected (%)	Total	Infected (%)	Not infected (%)				
15–25	25	2 (8%)	23 (92%)	41	34 (82.93%)	7 (17.07%)	66	30 (45.45%)	36	54.55%
26–36	31	4 (12.90%)	27 (87.10%)	43	39 (90.69%)	4 (9.30%)	74	31 (41.89%)	43	58.11%
37–47	40	9 (22.5%)	31 (77.5%)	34	20 (58.82%)	14 (41.18%)	74	45 (60.81%)	29	39.19%
≥48	52	37 (71.15%)	15 (28.85%)	22	10 (45.45%)	12 (45.45%)	74	27 (36.49%)	47	63.51%

Statistically significant at *P* < 0.05.

**Table 3:** Distribution frequency of isolated bacterial uropathogens.

Bacterial pathogens	Frequency (%)	*χ* ^2^ value	*P* value
*Escherichia coli *	66 (42.58%)	30.000	0.224 (*P* < 0.05, Significant)
*Klebsiella pneumoniae *	29 (18.71%)		
*Pseudomonas aeruginosa *	20 (21.90%)		
*Proteus *spp.	14 (9.03%)		
*Enterobacter *spp.	11 (7.10%)		
Total Gram negative	140 (90.32%)		
*Staphylococcus aureus *	15 (9.68%)		
Total Gram positive	15 (9.68%)		
Total Gram negative and positive	155 (100%)		

**Table 4:** Distribution of uropathogens in relation to sex and age of patients.

Uropathogens	Number	Age groups (in years)							
		15–25		26–36		37–47		≥48	
		Male	Female	Male	Female	Male	Female	Male	Female
*E. coli *	66	2 (3.03%)	14 (21.21%)	3 (4.55%)	11 (16.67%)	7 (10.61%)	5 (7.57%)	20 (30.30%)	4 (6.06%)
*K. pneumoniae *	29	—	11 (37.93%)	1 (3.45%)	7 (24.14%)	1 (3.45%)	2 (6.89%)	6 (20.69%)	1 (3.45%)
*P. aeruginosa *	20	—	2 (10.00%)	—	7 (35.00%)	1 (5.00%)	4 (20.00%)	4 (20.00%)	2 (10.00%)
*Proteus *spp.	14	—	1 (7.14%)	—	4 (28.57%)	—	5 (35.71%)	3 (21.43%)	1 (7.14%)
*Enterobacter *spp.	11	—	3 (27.27%)	—	5 (45.45%)	—	—	2 (18.18%)	1 (9.09%)
*S. aureus *	15	—	3 (20.00%)	—	5 (33.33%)	—	4 (26.67%)	2 (13.33%)	1 (6.67%)
Total	155	2	34	4	39	9	20	37	10

**Table 5:** Resistant and susceptibility rates (%) for isolated uropathogens.

Antibiotics	*E. coli* (66)		*K. pneumonia* (29)		*P. aeruginosa* (20)		*Proteus *spp. (14)		*Enterobacter *spp. (11)		*S. aureus* (15)	
	%R	%S	%R	%S	%R	%S	%R	%S	%R	%S	%R	%S
CIP	69.69	30.30	79.31	20.69	5	95	35.71	64.29	18.18	81.82	33.33	60.00
MOX	56.06	40.91	58.62	41.38	NT	NT	42.86	50.00	9.09	90.91	60.00	40.00
OFL	40.91	56.06	6.89	82.76	15	85	57.14	42.86	0	100	13.33	86.67
SPR	37.88	56.06	51.72	48.28	100	0	21.43	78.57	0	100	6.67	93.33
LEV	27.27	66.67	10.34	89.66	40	60	14.29	85.71	0	100	26.67	73.33
NAL	90.91	7.58	65.52	34.48	85	20	64.29	35.71	18.18	72.73	100	0
GTX	60.61	39.39	20.69	79.31	NT	NT	50.00	50.00	9.09	81.82	33.33	66.67
TOB	96.97	3.03	68.97	31.03	60	40	7.14	85.71	27.27	72.73	20.00	73.33
AMK	9.09	90.91	17.24	79.31	95	0	28.57	71.43	18.18	81.82	80.00	20.00
GET	68.18	30.30	34.48	65.52	10	90	21.43	71.43	9.09	90.91	13.33	80.00
CTZ	78.79	18.18	79.31	13.79	65	35	78.57	21.43	45.45	54.55	46.67	53.33
CTX	87.88	10.61	37.93	62.07	90	5	14.29	78.57	9.09	90.91	100	0
CFX	53.03	46.97	20.69	79.31	95	5	92.86	0	54.55	36.36	0	93.33
IMP	0	98.48	75.86	24.14	5	95	0	100	0	100	0	100
MRP	4.55	95.45	13.79	86.21	0	100	0	100	0	100	20.00	80.00
NTF	25.76	74.24	62.07	37.93	90	10	57.14	42.86	81.82	18.18	6.67	86.67
NTL	15.15	84.85	27.59	74.41	20	80	85.71	14.29	36.36	62.64	6.67	93.33
COT	84.85	15.15	34.48	65.52	5	85	28.57	64.29	45.45	54.55	53.33	40.00

CIP: ciprofloxocin; MOX: moxifloxacin; OFL: ofloxacin; SPR: sparfloxacin; LEV: levofloxacin; NAL: nalidixic acid; GTX: gatifloxacin; TOB: tobramycin; AMK: amikacin; GET: gentamycin; CTZ: ceftazidime; CTX: cefotaxime; CFX: ceftriaxone; IMP: imipenem; MRP: meropenem; NTF: nitrofurantoin; NTL: netillin; COT: co-trimoxazole; R: resistant; S: sensitive; NT: not Tested.
